# ANNOUNCEMENT / NOUVELLE

**DOI:** 10.29173/jchla29747

**Published:** 2023-12-01

**Authors:** Christine Neilson

## Winnipeg is the place to be this June!

The CHLA/ABSC conference planning committee is hard at work, preparing another outstanding conference experience. The conference is being held at the Hotel Delta Winnipeg from June 11-14, 2024. This year’s theme is Disruptive & Diverse, highlighting the transformative power of diverse perspectives and innovative disruptions, while acknowledging the dynamic challenges and opportunities in health science libraries today. The draft program-at-a-glance is available for you to get a sense of what is happening and when. Submissions are accepted online until **January 15, 2024, 11:59 pm CDT**.

We have a unique opportunity this year: True North Science Bootcamp (TNSBC) is being offered in Winnipeg on the University of Manitoba Campus in the days immediately prior to the CHLA/ABSC conference. We will be holding a joint reception at the closing of TNSBC and the opening of CHLA 2024 in the evening of Tuesday, June 11th – it will be a great way to make some new connections in the broader library community. If you’re looking to maximize PD travel funds by attending two conferences with one plane ticket, be sure to check out what’s on offer at TNSBC this spring and see if this event is right for you.

If you’re interested in seeing the sights while you’re in town, the Delta is within walking distance of the Winnipeg Art Gallery, The Canadian Museum for Human Rights, the Forks National Historic Site, and Shaw Park, home to the Winnipeg Goldeyes baseball team. If you’re willing to go further afield, other major attractions in the city include the St. Boniface Museum, Assiniboine Park Zoo, The Leaf at Assiniboine Park, and the FortWhyte Alive nature centre. See the “Welcome to Winnipeg” section of the conference website to get some ideas on how you might spend a few days in the city.

We look forward to welcoming you to Winnipeg this spring!

**Christine Neilson**

*2024 Conference Planning Committee, Publicity*

## Winnipeg est l'endroit où il faut être en juin!

Le comité de planification du congrès de l'ABSC / CHLA travaille d'arrache-pied à la préparation d'un autre congrès exceptionnel. Le congrès se tiendra à l'hôtel Delta Winnipeg du 11 au 14 juin 2024. Le thème de cette année est Disruptif et diversifié, soulignant le pouvoir de transformation des perspectives diverses et des perturbations innovantes, tout en reconnaissant les défis dynamiques et les opportunités dans les bibliothèques des sciences de la santé aujourd'hui. L’aperçu du programme provisoire est disponible pour vous donner une idée de ce qui se passe et quand. Les soumissions sont acceptées en ligne jusqu'au **15 janvier 2024, 23h59 HAC**.

Nous avons une opportunité unique cette année : le camp d'entraînement scientifique True North (TNSBC) est offert à Winnipeg sur le campus de l'Université du Manitoba dans les jours précédant immédiatement le congrès de l'ABSC / CHLA. Nous organiserons une réception commune à la clôture du TNSBC et à l'ouverture de l'ABSC 2024 dans la soirée du mardi 11 juin - ce sera une excellente façon d'établir de nouvelles relations dans la communauté élargie des bibliothèques. Si vous cherchez à maximiser les fonds de voyage de développement professionnel en participant à deux conférences avec un seul billet d'avion, assurez-vous de vérifier ce qui est offert à TNSBC ce printemps et de voir si cet événement est pour vous.

Si vous souhaitez visiter la ville, le Delta se trouve à quelques pas de la Winnipeg Art Gallery, du Musée canadien des droits de l'homme, du site historique national de La Fourche et du Shaw Park, où joue l'équipe de base-ball des Winnipeg Goldeyes. Si vous êtes prêt à aller plus loin, d'autres attractions majeures de la ville comprennent le musée de Saint-Boniface, le zoo du parc Assiniboine, The Leaf au parc Assiniboine et le centre de la nature FortWhyte Alive. Consultez la section Bienvenue à Winnipeg du site web de la conférence pour avoir quelques idées sur la façon dont vous pourriez passer quelques jours dans la ville.

Nous nous réjouissons de vous accueillir à Winnipeg ce printemps!

**Christine Neilson**

*Comité de planification de la conférence 2024, publicité*

